# Missing value imputation for microarray gene expression data using histone acetylation information

**DOI:** 10.1186/1471-2105-9-252

**Published:** 2008-05-29

**Authors:** Qian Xiang, Xianhua Dai, Yangyang Deng, Caisheng He, Jiang Wang, Jihua Feng, Zhiming Dai

**Affiliations:** 1 Department of Electronics & Communications Engineering, School of Information Science and Technology, Sun Yat-Sen University, 135 West Xin'gang Road, Guangzhou, PR China

## Abstract

**Background:**

It is an important pre-processing step to accurately estimate missing values in microarray data, because complete datasets are required in numerous expression profile analysis in bioinformatics. Although several methods have been suggested, their performances are not satisfactory for datasets with high missing percentages.

**Results:**

The paper explores the feasibility of doing missing value imputation with the help of gene regulatory mechanism. An imputation framework called histone acetylation information aided imputation method (HAIimpute method) is presented. It incorporates the histone acetylation information into the conventional KNN(*k*-nearest neighbor) and LLS(local least square) imputation algorithms for final prediction of the missing values. The experimental results indicated that the use of acetylation information can provide significant improvements in microarray imputation accuracy. The HAIimpute methods consistently improve the widely used methods such as KNN and LLS in terms of normalized root mean squared error (NRMSE). Meanwhile, the genes imputed by HAIimpute methods are more correlated with the original complete genes in terms of Pearson correlation coefficients. Furthermore, the proposed methods also outperform GOimpute, which is one of the existing related methods that use the functional similarity as the external information.

**Conclusion:**

We demonstrated that the using of histone acetylation information could greatly improve the performance of the imputation especially at high missing percentages. This idea can be generalized to various imputation methods to facilitate the performance. Moreover, with more knowledge accumulated on gene regulatory mechanism in addition to histone acetylation, the performance of our approach can be further improved and verified.

## Background

DNA microarray technology can simultaneously measure the mRNA levels of thousands of genes under certain experiments. It gives a global overview of gene expression profiles in particular cells or tissues, so it has become one of the most prominent tools in functional genomics research. The analysis of gene expression profiles is aim to discover a broad range of biological disciplines and predict a clinical state or other effects such as cancer classification and relevant genes identification, mechanism investigation and cancer prognosis [1-7]. Though playing crucial roles in these studies, the existing multivariate analysis methods for expression profile data have been greatly negatively affected by the high percentage of missing values, e.g. hierarchical clustering and the support vector machine classifier [8,9]. Moreover, many analysis methods such as principal component analysis (PCA), singular value decomposition (SVD) and generalized SVD (GSVD) cannot be applied to datasets with missing values [10-12].

Missing values often occurred due to various reasons, such as insufficient resolution, image corruption, dust or scratches on the slide and etc., however none dominated. Generally, microarray datasets are estimated to have more than 5% missing values and up to 90% of genes are affected [13,14]. As a result, although often disregarded, the missing value imputation is essential to minimize the detrimental effect of missing values on the microarray data analysis.

Certainly, one strategy to validate the analysis method of the microarray data with missing values is to repeat the experiments, and obviously it is very expensive and time consuming [15,16]. There are also several simple ways to handle missing values, e.g. removing the genes with missing values from further analysis, replacing missing values by zeros, or filling the missing values with the row or column averages available [17,18]. These approaches are not optimal because they did not consider the correlation of the data, which encouraged the development of more refined missing value procedures that tried to exploit the data relationships by using the information available in the whole dataset [19].

Therefore, many imputation methods have been proposed since 2001, such as *k*-nearest neighbour(KNN), singular value decomposition(SVD), local least square(LLS), Bayesian principal component analysis(BPCA), Gaussian mixture clustering (GMC), collateral missing value estimation(CMVE) and weighted nearest neighbors method(WeNNI)[14,16,20-23]. However all of the above methods are solely based on the gene expression datasets themselves and utilize nothing of the external microarray datasets or biological related information.

Integrative Missing Value Estimation method (iMISS) is the first to incorporate information of multiple reference microarray datasets to improve missing data imputation [24]. However, it is difficult to find various datasets and even more difficult to find a set of genes frequently show expression similarity to the target gene over multiple datasets. Meanwhile, GOimpute is the first algorithm that exploits the functional similarities embedded in the GeneOntology(GO) databases along with the expression similarities to facilitate the neighbor gene selection [13]. It outperformed KNN at high missing percentages, while due to the limitation of the number and accuracy of the gene functions annotated in GO databases, GOimpute failed to improve the LLS.

As summarized in Table 1, missing value is a common problem that has to be addressed even for more recent studies [25,26]. Furthermore, there are many genes with high missing percentages. In this case, for genes with many missing values, few values are remained to determine how the gene is correlated with other genes in the dataset, which leads to less accurate estimates. It is well known that gene expressions in eukaryotic cells are concertedly regulated by transcription factors and chromatin structure [27]. The fundamental repeating unit of chromatin is nucleosome, which consists of approximately 146 base pairs of DNA wrapped around an octamer of four core histones (H2A, H2B, H3, and H4) [28]. As mentioned in [29-31], histone acetylation may alter chromatin structure and provide binding surfaces for transcription factors. Therefore, the transcription factor activity is highly regulated by the histone acetylation states in chromatin. Consequently, the gene expression profiles are regulated by the histone acetylation states [32]. Intuitively, this means that one of the important prerequisites for gene co-expression is the similar acetylation state in their chromatin. With this idea in the mind, the use of available acetylation information will somehow contribute to the selection of neighbour genes especially for microarrays with high missing percentages.

**Table 1:** Number of genes with different percentages of missing values in the datasets

Datasets	Number of arrays		Number of genes		Number of genes with specific missing percentages (%)				
			
	*N*	*N'*	*L*	*L'*	0~5	5~10	10~15	15~20	>20
Histone	24	24	6120	3913	1074	98	25	749	261
Gasch	173	44	6153	2990	2597	317	139	55	54
Calcineurin	24	24	6586	3367	0	1102	312	162	420
Sp.elu	14	14	6178	5766	0	296	11	0	105
Sp.alpha	18	18	6178	4489	0	1183	282	65	159
Diauxic	7	7	6068	5875	0	0	0	191	2

*N *and *L *are the number of experimental conditions and genes in the original dataset respectively. *N' *and *L' *are the number of experimental conditions and genes in the dataset after removing the genes with missing values.

In this paper, we proposed an imputation framework called HAIimpute to integrate histone acetylation information to improve the estimation accuracy of the missing values in gene expression datasets. We applied HAIimpute to both LLS and KNN, namely, llsHAI and knnHAI. The performance of HAIimpute is compared with LLS, KNN and GOimpute over various well characterized *Saccharomyces cerevisiae *(yeast) datasets outlined in Table 2. These datasets include time-series datasets, non-time series dataset and mixed-type dataset (comprises both time and non-time series). It seems that our approaches showed a significant and consistent improvement compared to KNN and LLS while GOimpute did not achieve [13]. In addition, the application of an iterative process to HAIimpute further improves the accuracy.

**Table 2:** Summary of the datasets

Datasets	Title	Microarray Platform			Experimental type
			
		Type	Organism	Fabricate protocol	
Histone	Meneghini 2003 [25]	spot cDNA	yeast	described by [34]	mutant/wild-type (non-time series)
	Kobor 2004 [26]	spot cDNA	yeast	described by [34]	mutant/wild-type (non-time series)
Gasch	Gasch 2000 [33]	spot cDNA	yeast	described by [34]	stress response (non-time series)
Calcineurin	Yoshimoto 2002 [36]	spot cDNA	yeast	described by [34]	logical set (mixed type)
Sp.elu	Spellman 1998 [35]	spot cDNA	yeast	described by [34]	cell cycle (time series)
Sp.alpha	Spellman 1998 [35]	spot cDNA	yeast	described by [34]	cell cycle (time series)
Diauxic	DeRisi 1997 [34]	spot cDNA	yeast	custom	diauxic shift (time series)

## Results

### Datasets

Many studies about the gene expression and histone acetylation have been conducted extensively in the yeast, so it is chosen to be the model organism in the paper to test the imputation accuracies of the methods [25,26,30,33-39]. Datasets analyzed in this study include those for gene expression and histone acetylation.

### Gene expression data

Several cDNA microarray datasets of yeast were downloaded from the publicly available website [40]. The datasets are chosen to represent diverse types of experimental conditions including time series, non-time series and mixed-type. At the same time, in order to evaluate the performance of the methods under various numbers of experimental conditions, datasets with small and large number of conditions are selected. So that results are likely to hold for cDNA microarray datasets in general. The summary of the experimental details of the datasets is shown in Table 2. The first dataset (Histone) is a combined dataset from the experiment data of two studies[25,26]. The study of [25] is trying to indicate the critical role of histone variant H2A.Z on protecting euchromatin and affecting gene expression, while the study of [26] is about the deposition of histone variant H2A.Z by a multi-subunit protein complex and the effects on global gene expression. The combined dataset contains 24 different non-time series experimental conditions. Another non-time series dataset is the study of response to environmental changes in yeast from [33]. It contains 6152 genes and 173 experimental conditions that have time-series of specific treatments. After removing all the experimental conditions with more than 8% missing entries, we select all genes without the missing values. Among the resulting 2990 genes, 44 experimental conditions are randomly selected and rearranged to construct a non-time series subset of the whole original dataset. The third dataset (Diauxic) is a temporal gene expression dataset studying metabolic shift from fermentation to respiration in yeast. It is a time-series dataset from [34] and contains 7 sampling points for each genes. Another two time-series datasets are the alpha part(SP.alpha) and the elutriation part(SP.elu) of Spellman datasets which focus on the identification of yeast cell cycle genes [35]. They have 18 and 14 sampling points respectively. These three time-series datasets are also chosen to evaluate the influence of different number of the sampling points on the performance of the methods. The last dataset is Calcineurin dataset which focus on gene expression in calcineurin/crz1p signal pathway [36]. It contains 4498 gene expression profiles and 24 experimental conditions with both time-series and non-time series.

### Histone Acetylation Data

Since histone acetylation is one of the most important modifications of the histone proteins, it mainly affects gene transcription in the following two major pathways [37,41]. On one hand, histone acetylation may alter the folding properties of the chromatin fibre and influence the binding of transcription factors, thereby modulating the accessibility of target binding sites in the DNA through structural properties changes [42]. On the other hand, histone acetylation will expose specific binding surfaces for the recruitment of transcription factors, so as to affect the gene activity and regulate the gene expression. Therefore, such modifications can always be maintained through cell division and propagated to proximal nucleosomes by positive-feedback mechanisms. The activation of a transcription factor can also be temporally and spatially transmitted through chromatin [43,44].

Nowadays, the rapidly accumulated histone acetylation information in public databases have provided a genome-wide map of genomic regions that are enriched or depleted of histone acetylation [38,39]. Also available are datasets measuring the relative association of the histone proteins themselves (H2A, H2B, H3 and H4) with DNA [30,37]. The sites of acetylation include at least four highly conserved lysines in histone H4(K5, K8, K12 and K16), five in histone H3(K9, K14, K18, K23 and K27), as well as less conserved sites in histone H2A and H2B [31]. Various histone acetylatransferases and deacetylases show distinct specificities for different acetylation sites, which generate unique histone acetylation patterns that involve combinations of differently acetylated sites [45,46].

It is reported in [37] that unique patterns of histone acetylation are each enriched for genes that have significant mRNA expression coherence under a wide range of experimental conditions in addition to the same conditions in which acetylation data were obtained. Furthermore, the genes that share common acetylation patterns are also enriched for specific transcription factor binding sites and common DNA motifs. All these phenomena verified that acetylation patterns play fundamental roles in diverse chromatin-based processes to coordinate the regulation of related genes [47]. The histone acetylation data used in this paper were from the study of [37], where genome wide histone acetylation levels at 11 sites for both intergenic regions (IGRs) and open reading frames (ORFs) were measured using ChIP-chip and the acetylated DNA was hybridized against the genomic DNA on microarrays.

### Models for missing values

In order to evaluate the performance of missing value imputation, genes that contain missing values are removed from the original expression datasets to construct the complete datasets. Based on these datasets, two different missing value models are designed to introduce artificial missing values in percentage-based way from 1% to 20%.

### Random model of missing values

In this model, the datasets with missing values were constructed by removing a specific percentage of the values (between 1% and 20%) from the datasets randomly and independently. Each entry in the dataset has the equal probability to be a missing value.

### Burst model of missing values

From the observation of the actual datasets in Table 1, many datasets are enriched of genes with high percentages of missing values. Furthermore, most of these missing values occur in a successive way. Therefore, we design a burst model of missing values in order to simulate this realistic missing pattern caused by experimental systematic errors.

If denoting the microarray dataset to be a N × L matrix

(1) **G **= (**g**_1 _⋯ **g**_*L*_) ∈ ℜ^*N*×*L*^

where column **g**_*i *_∈ ℜ^*N*×*L *^denotes the expression of gene *i *(1 <*i *≤ L) under *N *different experimental conditions.

In the burst model, some artificial missing values are generated in series, namely, a burst. A burst of missing value is defined as a length of *B *successive missing elements in a column of **G**. If the missing percentage is set to be *p *between 1% and 20%, the total number of the missing values is *L *× *N *× *p*, the number of genes with burst of missing values is $\left\lceil \frac{L\times N\times p}{B}\right\rceil$. Therefore, if the dataset **G**, missing percentage *p*, as well as the value of *B *are given, we can randomly sample some genes from **G **and randomly assign burst of missing values to each of these genes.

### Evaluation measures

Each method was applied to recover the introduced missing values for every dataset. The performance was evaluated by calculating the normalized root mean squared error (NRMSE) [24]. Assume $\hat{G}$ is the imputed microarray matrix, then the NRMSE for the predictor was defined as:

(2) $$\text{NRMSE}=\sqrt{\frac{mean[{\left(G-\hat{G}\right)}^{2}]}{mean[G^{2}]}}$$

where the mean is calculated over missing values in the whole matrix. **G **is known because missing values are artificial. From (2), NRMSE approaches its minimum value 0.0 if the imputation is accurate. Otherwise, if the NRMSE becomes much larger, the imputation is poor.

For more careful evaluation of imputation efficiency, Pearson correlation coefficients were calculated in addition to NRMSE between imputed dataset and complete dataset for each gene with missing values [48]. We take the mean value of these correlation coefficients as another evaluation measure. Based on this measure, we could find how the data structure of each gene was preserved after imputation with different imputation methods. The larger the correlation coefficient is, the better the relationship is preserved between imputed gene expression and complete gene expression, which is a very important property for gene when doing some gene selection analysis.

### Parameters setting

The values of the neighbourhood size *k *for KNN, LLS, GOimpute and HAIimpute methods are important model parameters for obtaining high performances. In accordance with the study of Kim 2005, there is no theoretical result to determine these parameters optimally [20]. Therefore, heuristic procedures are used to estimate parameter *k *in this paper.

### Selection of the neighbourhood size *k*

It was reported in [16,20] that the best results of KNN-based method are obtained by setting *k *between 10 and 20, while the best results of LLS-based method are obtained by setting *k *more than 100. Therefore in order to reduce the complexity, we used KNN imputation method to select the optimal values of *k *for KNN, GOKNN and knnHAI by evaluating the imputation accuracy of KNN in the range of *k *= 5 ~ 40. Similarly, we used LLS imputation method to select the optimal values of *k *for LLS, GOLLS and llsHAI by evaluating the imputation accuracy of LLS in the range of *k *= 60 ~ 200. We generated 50 random missing datasets for each test run at each missing percentage. The results are presented in Additional files 1 and 2 for KNN and LLS respectively. We observed that 10 neighbours were enough in most cases of each dataset for KNN, while 150 neighbours are enough in most cases for LLS, which is consistent with the observations of [13,20]. Thus the value of *k *= 10 and *k *= 150 were used in the following test runs.

### Selection of the parameter *λ*

For HAIimpute methods, parameter *λ *is also an important factor (See Methods). It is a positive weight between 0 and 1, which determines the contribution of acetylation patterns to the final imputation for missing values. Because acetylation patterns have different effects in various datasets, the value of *λ *was estimated by a training procedure for each dataset. Firstly, genes in each dataset will be divided into two subsets randomly. One subset consists of 20% of the genes which is regarded as the training subset, while another subset consists of 80% of the genes which is regarded as the testing subset. So the training and testing procedure were done on totally separate and different subsets. Then in the training procedure, 50 independent simulations were executed on the training subset genes to select the optimal *λ *value which produced the minimum NRMSE at each miss percentage. Finally, in the testing procedure, this optimal *λ *will be used for the actual missing value imputation for the testing subset with 80% of the genes in the corresponding dataset. In this case, 100 independent simulations were executed for various missing percentages. So only the information in training subset genes was allowed to be used in the training procedure and there is no information leakage from training to testing. The simulation results are shown in Additional files 3 and 4 for knnHAI and llsHAI respectively.

### NRMSE Performance with respect to percentage of missing values

The NRMSE performance of the HAIimpute methods (including both knnHAI and llsHAI) are evaluated over six different datasets and compared with three other approaches, i.e., KNN, LLS and GOimpute (including both GOKNN and GOLLS). The neighbourhood size *k *in KNN, GOKNN and knnHAI were preset as 15 in each test run according to the simulation results in Additional file 1.

Similarly for LLS, GOLLS and llsHAI, *k *will be set at 150 without loss of generality. In order to obtain results unbiased with regard to the missing part of the datasets, 100 independent and random simulation rounds were executed for each dataset under various missing percentages. The missing values are generated by the two different models described above.

The source code of GOimpute was kindly presented by the author [13]. It used an adaptive selective procedure to select the weight parameter that controls how much of the GO information is used in the imputation process, so the weight parameter is optimized in each dataset and at each missing value rate automatically. As for conventional KNN and LLS, they have no such parameters to be tuned. Therefore, the optimal results of each method are used in performance comparisons. In this case, both the optimization of *λ *on the training subset (20% of the genes) and the testing of the imputation method on the remaining subset (80% of the genes) were carried out under the same missing value model.

### NRMSE Performance under random model of missing values

**Figure 1:**
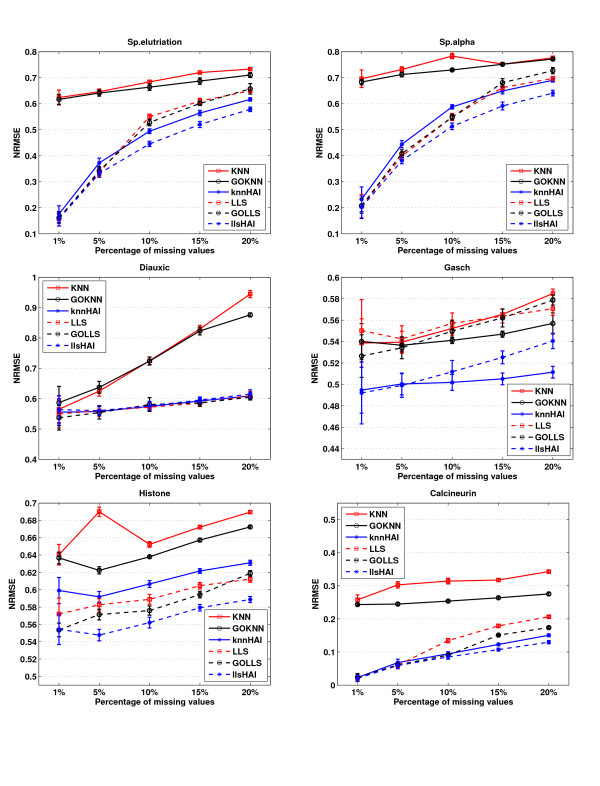
**NRMSE performance under random model of missing values**. Comparisons of NRMSE performances for KNN, LLS, GOKNN, GOLLS, knnHAI, and llsHAI in six datasets under random model of missing values. The horizontal axis is the varying range of missing percentages from 1% to 20%. The vertical axis is NRMSE of 100 independent and random test runs for each method. The knnHAI method outperforms KNN and GOKNN, while llsHAI mostly outperforms LLS and GOLLS. Generally, llsHAI performs best in most cases.

The missing values are equally distributed if they are introduced with a random model. Figure 1 shows the NRMSEs of the methods for each dataset in the presence of different percentages of equally distributed missing values. From Figure 1, we observed that the HAIimpute methods produce more accurate imputation results than KNN, LLS and GOimpute methods in almost all the datasets, especially at higher missing percentages.

SP.elu, SP.alpha and Diauxic are three time-series datasets with different number of sampling points. The performance of the knnHAI is better than that of the conventional KNN and GOKNN across all the missing percentages in these three datasets. For llsHAI, specifically, in the datasets of SP.elu and SP.alpha, if missing percentage is below 5%, the NRMSE of the llsHAI is comparable with, if not better than those of LLS and GOLLS. If missing percentage is above 5%, llsHAI shows good performance and outperforms LLS and GOLLS. This indicates HAIimpute methods can help to select more strongly correlated genes than other methods in these two datasets. While an interesting phenomenon in the dataset of Diauxic is that the improvement of llsHAI is not obvious, which is similar to what was observed in the GOimpute study by Johannes [13].

As claimed by [16], Gasch is the most challenging dataset, where clear expression patterns are often absent. Gasch is a non-time series dataset. Figure 1 shows that the llsHAI and knnHAI consistently achieve better results over the whole range of test missing percentages in all the cases. One interesting observation is that knnHAI yields smaller NRMSE than llsHAI at higher missing percentages. In Gasch dataset, experimental conditions are randomly selected from the original dataset, so the correlated relationships among the experimental conditions are not as strong as that in the original dataset. In order to obtain results unbiased with regard to the selection of the experimental conditions from the datasets, we have independently and randomly selected the experimental conditions for 10 times and the results are similar and consistent. Since HAIimpute methods can provide relative more accurate values for the missing values, more proper neighbour genes can be selected and benefit KNN. While since LLS is more sensitive on the local similarity structure of the dataset than KNN, the performance of LLS is not improved so much because of the loss of the similarity among the experimental conditions.

Another non-time series dataset is Histone dataset relevant to histone variant H2A.Z and its role in gene expression, which involves much more complex regulatory mechanisms. Therefore, this dataset is more difficult for missing value imputation. From the performances of the methods in this dataset shown in Figure 1, the accuracies of the HAIimpute methods are superior over all test missing percentages. The Calcineurin dataset studied in [36], is a mixed-type expression dataset regulated by the Calcineurin/Crz1p signaling pathway in yeast. The comparison results in Calcineurin dataset show that the HAIimpute methods performed more robustly as the percentage of the missing values increase. Particularly, when the missing value percentage is below 10%, the llsHAI method gets comparable estimating ability with the GOLLS. When the missing value percentage exceeds 10%, the NRMSE of LLS and GOLLS become large while llsHAI is more robust. For knnHAI, it performs better than KNN and GOKNN at all the missing percentages.

### NRMSE Performance under burst model of missing values

**Figure 2:**
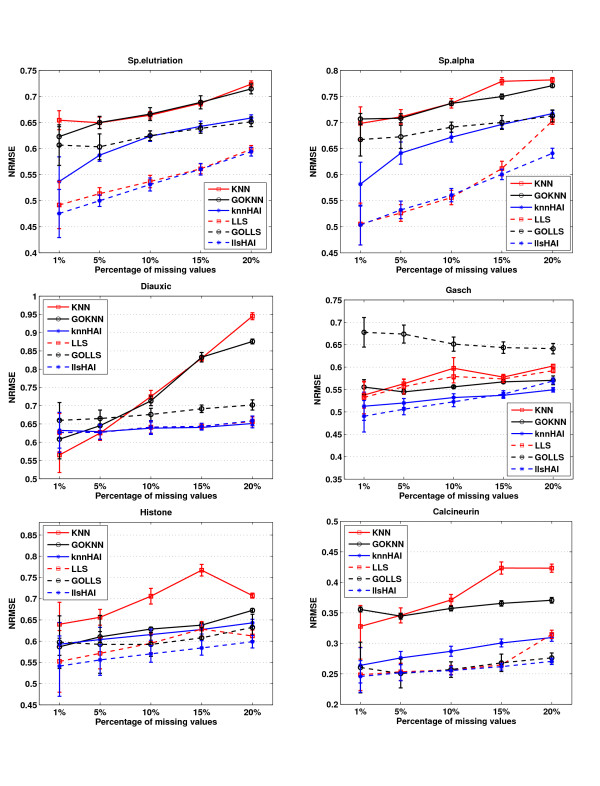
**NRMSE performance under burst model of missing values**. Comparisons of NRMSE performances for KNN, LLS, GOKNN, GOLLS, knnHAI, and llsHAI in six datasets under burst model of missing values. The legends are the same as Figure 1. The HAIimpute methods are more robust than GOimpute methods in this case. The knnHAI method outperforms KNN and GOKNN, while llsHAI outperforms LLS and GOLLS in most cases.

If the missing values are introduced with a burst model, the missing values are distributed unequally. The comparisons of HAIimpute methods with KNN, LLS and GOimpute methods in this case are shown in Figure 2 for each dataset under various missing percentages. We observed that the HAIimpute methods still produce more accurate results than the conventional KNN, LLS and GOimpute methods in the case of burst model of missing values.

Comparing with the results in random model, it seems that the performances of HAIimpute methods are more robust than those of conventional KNN, LLS and GOimpute methods in the burst model of missing values. Especially in the datasets of Gasch, Histone and Calcineurin, the performance improvements of HAIimpute methods over conventional methods are still significant, while the performances of GOimpute are greatly degraded and even worse than those of the conventional KNN and LLS. As for three time-series datasets of SP.elu, SP.alpha and Diauxic, knnHAI can still performs much better than KNN and GOKNN, while the improvement of llsHAI is not obvious as they have performed in the case of random missing model. In order to test the cases that the underlying missing value models are not known in practice, another experiment is done to test whether the missing value model has an impact on the imputation results. In this case, the random model is used in the training phase to optimize the parameter *λ*, while the burst model is used in the testing phase. While for GOimpute, we used its optimal results for comparison. The results in Additional file 5 are very similar with the results in Figure 2, which showed that the HAIimpute method is robust even though we do not know the underlying missing value models.

### Pearson correlation between imputed dataset and original complete dataset

**Figure 3:**
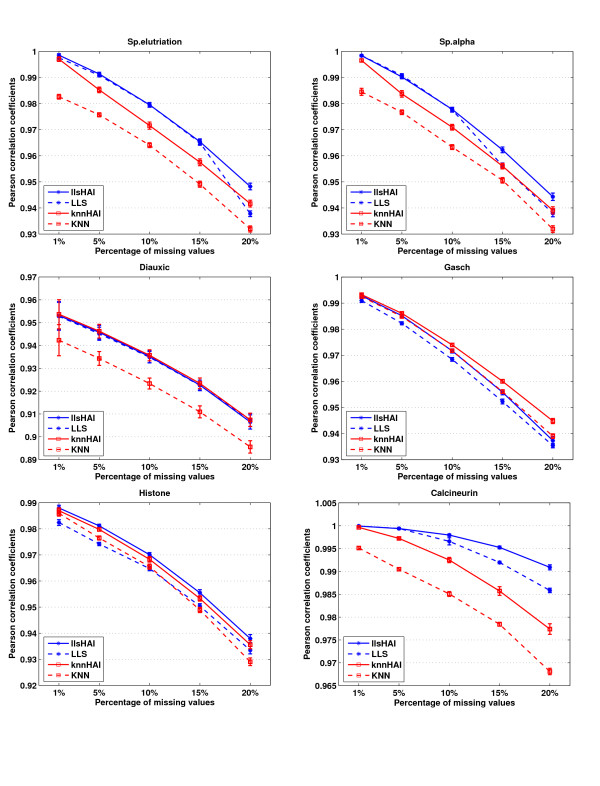
**Pearson correlation between imputed dataset and original complete dataset**. Comparisons of Pearson correlation coefficients for KNN, LLS, knnHAI and llsHAI in six datasets under random model of missing values. The horizontal axis is the varying range of missing percentages from 1% to 20%. The vertical axis is Pearson correlation coefficients of 100 independent and random test runs for each method. We observed that the results are consistent with the NRMSE performances in Figure 1 across the whole test missing percentages. The genes imputed by llsHAI are most correlated with the original complete genes while the genes imputed by knnHAI are more correlated with the original complete genes than those imputed by KNN.

The Pearson correlation coefficients between imputed dataset and original complete dataset are calculated for each gene with missing values. This is another evaluation method which is to measure the correlated relationship between the imputed dataset and the original complete dataset with different imputation methods. The performances of HAIimpute methods are compared with conventional KNN and LLS in terms of this measure. The neighbourhood size *k *in KNN and knnHAI was set the same value as before. So is the *k *value for LLS and llsHAI. We execute 100 independent and random simulation rounds for all six datasets under various missing percentages and the missing values are generated by the random model described above. The comparison results are shown in Figure 3. The results are consistent with the NRMSE performances in Figure 1 across the whole test missing percentages. Specifically, the datasets imputed by llsHAI are most correlated with the original complete datasets while the datasets imputed by knnHAI are more correlated with the original complete datasets than those imputed by KNN.

### Influence of used histone acetylation patterns

Another important factor that influences the performance of the HAIimpute methods is how to use the histone acetylation patterns. For the accumulated histone acetylation data (See Datasets), the acetylation level of 11 acetylation sites for IGRs and ORFs are clustered to generate different acetylation patterns (See Methods). According to the published results of [37], there are 53 and 68 clusters which are identified to formulate the corresponding acetylation patterns for IGRs and ORFs respectively. For each dataset, genes which share the same acetylation patterns are selected and mean expressions of these genes are computed to form the corresponding pattern expressions (See Methods). In the proposed HAIimpute methods, these pattern expressions will be exploited to facilitate the imputation.

Here we test the NRMSE performances of HAIimpute methods if using acetylation patterns on different genomic regions, i.e., IGRs or ORFs. Meanwhile, in order to reduce the computational complexity, the methods select only part of the acetylation patterns for imputation. Therefore the performances when using different quantity of acetylation patterns are given for comparison. These were investigated in SP.elu dataset under different missing percentages and the results are presented in Additional file 6. For each case, we simulated 50 times of independent and random selection of the acetylation patterns for imputation.

We were surprised to find that the overall performances of HAIimpute methods using acetylation patterns on the ORFs seemed to be somehow better than those on the IGRs, especially for knnHAI. Another interesting observation is the behaviours of knnHAI and llsHAI: the imputation accuracy of knnHAI degrades markedly as the quantity of used acetylation patterns decreases. As for llsHAI, the influence is not so strong, but it does affect. In addition, when the quantity of used acetylation patterns is less than 23, the imputation accuracy will be degraded quickly if decrease the quantity of the used acetylation patterns. While more than 23, the imputation accuracy will be steady. This may caused by the fact that the regulation information associated with acetylation patterns may be redundant with the genomic sequence information, so that not all of the pattern expressions are significantly meaningful [27]. This observation may be partly explained by the results in [37].

### NRMSE Performance with respect to Iterative process

An iterative process to refine missing value estimates was implemented in llsHAI and knnHAI. The results in the time-series datasets (SP.alpha) and mixed-type dataset (Calcineurin) with respect to the number of iterations are shown in Additional file 7. In knnHAI, the number of iterations until convergence was in general smaller than three, whereas in llsHAI, one or two times of iterations were sufficient to achieve the convergence criterion (See Methods). From Additional file 7 we can see that the use of iterations in HAIimpute methods can refine the missing estimates in the presence of high missing percentages in these datasets because it minimises the bias introduced by the initial estimation step.

## Discussion

The paper proposed to incorporate the histone acetylation information into the conventional KNN and LLS methods, namely, knnHAI and llsHAI. It is well known that conventional methods are based on the assumption that if genes are co-expressed in some experimental conditions, they are assumed to have similar expressions in other experimental conditions with missing values [49]. This assumption is true if the genes share the same regulatory mechanism under different experimental conditions [50]. When the missing percentage is low, it is reliable for genes with missing values to find the co-regulated genes only based on the non-missing part. While in the case of high missing percentage, few values are remained and it is impossible to determine which genes are co-regulated with the target gene in the dataset. The functional similarity utilized in [13] may provide implicative information about gene expression in this case because co-expression genes are thought to have high probability to participate in the same biological functions. However, since histone acetylation provides a mechanism to straightforward coordinate the regulation of co-expressed genes, the integration of such regulatory information can provide more reliable evidence to assemble putative groups of co-expressed genes. The comparison results in Figures 1 and 2 demonstrated the performance improvements for the use of such acetylation information, and this also suggests that the histone acetylation information may be more highly correlated with the gene expression than that of functional similarity [51,52].

Since the integration of acetylation information gives relatively more accurate initial estimation for the missing values, it facilitates the selection of the neighbour genes based on Euclidean distance and contributes to the KNN. While LLS method is more dependent on the correlation between experimental conditions, the improvement of LLS is not as significant as KNN. Although the type of used histone acetylation (ORFs or IGRs) has no remarkable difference on the prediction accuracies, experimental results in Additional file 6 suggested that the incorporation of the acetylation information improves the imputation performance, especially at higher missing percentages. Therefore, it is recommended to use the acetylation information for the imputation when at higher missing percentages (e.g., > 10%). Actually, HAIimpute methods can be generalized to other imputation methods such as Bayesian principal component analysis or Gaussian mixture clustering that need more complete genes [14,21]. Comprehensive comparisons of different prediction methods are to be discussed in the future work.

Even though HAIimpute methods show good performances in our experiments, the algorithms also have some limitations. First, the histone acetylation information for many species or experimental conditions is not comprehensive, even for yeast, not all genes have available acetylation data, which limited the application of the methods. Nevertheless, we think that the HAIimpute methods introduced here open up a new angle to impute the gene expression from the gene regulatory mechanism itself. As for gene expression research, the eventual goal is to reveal the interior gene regulatory mechanisms rather than just predict the missing values themselves. Therefore, from gene regulation, various kinds of regulation factors, e.g., transcriptional factor binding sites, transcriptional factors and other histone modification data, could all be used in a concurrent way as long as the information to be integrated is well established and powerful. Second, we incorporate an iterative procedure to refine the imputation and it has higher computation cost than conventional KNN and LLS. In our experiments, the imputation for each dataset can be completed in no more than 30 seconds on a computer with 2.26 GHz CPU and 512 MB RAM using MATLAB. So we think the time cost is still acceptable in most cases. Furthermore, for some non-time series datasets, actually, we can do the imputation without iteration to save time since the improvement of iteration is not very significant. Third, although one burst model of missing values has been proposed in the paper to simulate the unequally distributed missing values, more proper alternatives to better describe the realistic distributions of missing values are increasingly needed.

## Conclusion

We have proposed an imputation framework, which can take advantage of the acetylation information to facilitate the imputation. The theoretical basis is that the acetylation states in chromatin provide a mechanism to straightforward coordinate the regulation of co-expressed genes. Therefore, the proposed methods repeatedly exploit the gene expression and the acetylation information in a combined way for the missing values imputation. Experimental results confirmed that HAIimpute methods consistently outperform the conventional KNN, LLS and GOimpute methods in various datasets, especially at higher missing percentages. Since the final imputations are still based on gene expressions, the idea can be generalized to various other imputation methods to facilitate the performance. Moreover, with more knowledge accumulated on gene regulatory mechanism, e.g., the mRNA decay, in addition to transcriptional factors and histone acetylation, the performance of our approach can be further improved and verified.

## Methods

**Figure 4:**
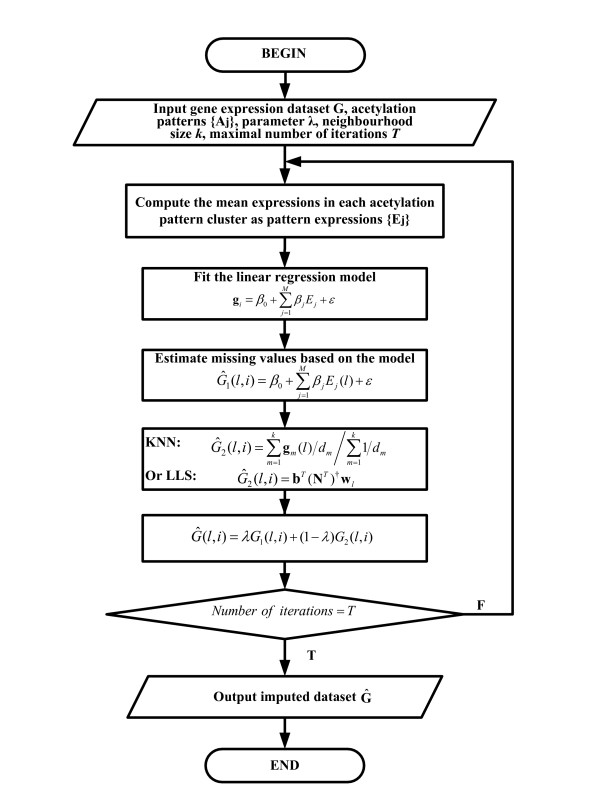
**Flowchart of the HAIimpute method**. The HAIimpute method mainly consists of the following steps: obtain the pattern expressions, fit the linear regression model, estimate the missing values based on the regression model, and impute the missing values based on the conventional methods, weighted combination and final decision.

The imputation procedure mainly consists of three steps. The first step is to preprocess the input data including gene expression datasets and histone acetylation datasets. In the second step, some acetylation patterns will be generated according to the combinations of 11 differently acetylated sites. Meanwhile, the genes in the histone acetylation datasets will be clustered according to different acetylation patterns. The final step is to impute the microarray dataset with the help of the acetylation. The flowchart of HAIimpute methods is shown in Figure 4.

### Pre-processing of the datasets

For the gene expression dataset **G**, the pre-process involves two steps. Firstly, in order to evaluate the estimation performance of the methods, we remove all the genes that contain missing values from the original datasets to construct the complete datasets. Then, for each dataset, the artificial missing values are introduced based on either two missing value models in percentage-based way from 1% to 20%. So the missing value in *l*-th location of *i*-th gene can be denoted as *G*(*l*, *i*) or **g**_*i*_(*l*).

For the acetylation datasets, the pre-process is mainly to remove some genes whose acetylation data have missing values.

### Gene Clustering based on Histone Acetylation Patterns

The 11 sites of acetylation can be combined to generate unique acetylation patterns that involve differently acetylated sites. Both intergenic regions (IGRs) and open reading frames (ORFs) are utilized to determine the genome-wide acetylation patterns. The method as described in [37] is used to find clusters of genes with similar acetylation patterns across the 11 residues. It was determined by the following procedures. Firstly, clusters of genes with similar acetylation patterns across the 11 residues are determined by conventional *k*-means algorithm. Then, each cluster was constrained to only include genes with a predefined minimal cut-off Pearson correlation coefficient on the acetylation levels (in our case, 0.75) and to have more than a minimal number of genes (in our case, 15). Finally, any cluster which does not satisfy this size constraint is reseeded from a random gene. In this way, 53 clusters for IGRs and 68 clusters for ORFs are identified respectively. So the value of *M *= 121. The clustering results can be denoted as $A_{O_{1}},\cdots A_{O_{n}}$ and $A_{I_{1}},\cdots A_{I_{n}}$ for ORFs and IGRs, respectively, where *O*_*n *_and *I*_*n *_are the maximal number of clusters for ORFs and IGRs. Each cluster consists of the genes which share common acetylation pattern and we denote *M *to be the total number of the acetylation patterns on IGRs and ORFs, namely, $M=A_{O_{n}}+A_{I_{n}}$. Therefore, by gene clustering based on acetylation levels, gene clusters characterized with different acetylation patterns are generated.

### HAIimpute methods

Now for all acetylation patterns $A_{O_{1}},\cdots A_{O_{n}}$ and $A_{I_{1}},\cdots A_{I_{n}}$, the mean expressions of all genes in these patterns are computed and denoted as *N *× 1 vectors {*E*_*j*_}, *j *= 1 ⋯ *M*, where *N *is the number of different experiments. Since the genes in each cluster may have missing values under some experiments, the average of the no-missing values was calculated for each experiment in the cluster. So only the no-missing parts of the gene expressions are used in calculate the mean expressions in this case. Because the mean expressions can represent some pattern expressions for genes which share the common acetylation pattern, we named it as pattern expression. Therefore, if the expression of gene **g**_*i *_and pattern expressions {*E*_*j*_}, *j *= 1 ⋯ *M *are available, these two sets of expressions are correlated by fitting a linear regression model of the form

(3) $$\begin{array}{cc} g_{i}=\beta_{0}+\sum_{j=1}^{M}\beta_{j}E_{j}+\varepsilon , & j=1\cdots M \end{array}$$

where *β*_0_,⋯, *β*_*M *_called the regression coefficients, *ε *represents the error term. Let ${\bar{g}}_{i}$ and ${\bar{E}}_{j}$ be the means of **g**_*i *_and *E*_*j*_, *S*_*g *_and *S*_*E *_be the standard deviations of **g**_*i *_and *E*_*j*_, respectively. The equation (3) stated in terms of standardized variables is

(4) $$\begin{array}{cc} {\tilde{g}}_{i}=\sum_{j=1}^{M}\eta_{j}{\tilde{E}}_{j}+\varepsilon^{\prime }, & j=1\cdots M \end{array}$$

where ${\tilde{g}}_{i}=(g_{i}-{\bar{g}}_{i})/S_{g}$ is the standardized version of the gene expression and ${\tilde{E}}_{j}=(E_{j}-{\bar{E}}_{j})/S_{E}$ is the standardized version of the pattern expressions. The estimated coefficients satisfy

(5) $$\begin{array}{cc} \beta_{j}=(S_{g}/S_{E})\eta_{j}, & \begin{array}{cc} j=1\cdots M & \beta_{0}=\bar{g}-\sum_{j=1}^{M}\beta_{j}{\bar{E}}_{j} \end{array} \end{array}$$

Without loss of generality, equation (4) in matrix form is

(6) $$\tilde{g}=\tilde{E}\eta +\varepsilon^{\prime }$$

where $\tilde{E}=({\tilde{E}}_{1},\cdots {\tilde{E}}_{M})$ is an *N *× *M *matrix of the standardized version of *M *pattern expressions under *N *different conditions. **η **is a *M *× 1 vector of regression coefficients. If there exist square matrices **Λ **and **V **satisfying

(7) $$\begin{array}{ccc} V^{T}({\tilde{E}}^{T}\tilde{E})V=\Lambda & \text{and} & V^{T}V=VV^{T}=I \end{array}$$

The matrix **Λ **is diagonal with the ordered eigenvalues of ${\tilde{E}}^{T}\tilde{E}$ on the diagonal, denoted by *λ*_1 _≥ *λ*_2 _≥ ⋯ ≥ *λ*_*M*_. The columns of **V **are the normalized eigenvectors corresponding to *λ*_1_,⋯,*λ*_*M*_. Since **VV**^*T *^= **I**, the regression model in equation (6) can be restated in terms of principal components (PCs) as

(8) **g **= **EVV**^*T*^**η **+ *ε *= **Cα **+ *ε*'

where **C **= **EV **and **α **= **V**^*T*^**η**.

The *M *columns **C**_1_,⋯,**C**_*M *_in matrix **C **are orthogonal and satisfy $C_{j}^{T}C_{j}=\lambda_{j}$ and $C_{i}^{T}C_{j}=0$ for *i *≠ *j*, which are referred to as PCs of the pattern expressions.

Since a small eigenvalue is an indicator of multicollinearity, which is associated with unstable estimated regression coefficients, the principal components regression approach is used here to reduce multicollinearity in pattern expressions for more accurate regression [53]. The reduction is accomplished by using less than full set of PCs to explain the variation in the target gene. As for the number of PCs used in regression, there is no universally agreed upon procedure in selecting the PCs to be included in the reduced model. We use the rule that only principal components associated with eigenvalues greater than 1.00 are of interest and kept for prediction [52]. If there are *p *PCs whose eigenvalues greater than 1.00, the regression model is

(9) **g **= **C**_1_*α*_1 _+ **C**_2_*α*_2 _+ ⋯ + **C**_*p*_*α*_*p *_+ *ε'*, *p *<*M*

Thus, the least square estimators for **α **can be expressed as

(10) **α **=(**C**^*T*^**C**)^-1^**C**^*T*^**g**

Then the principal components regression estimates of **η **corresponding to (4) can be computed by referring back to equation **η **= **Vα **and set the appropriate *α*'*s *to zero. The estimates of the regression coefficients **β **in equation (3) are obtained by substituting **η **in (5).

In this way, if **g**_*i *_has a missing value in *G*(*l*, *i*), the no-missing part ${\hat{g}}_{i}$ can be regressed over the pattern expressions deleting the *l*-th component. So the missing value *G *(*l*, *i*) can be estimated using the following equation

(11) $$\begin{array}{cc} {\hat{G}}_{1}(l,i)=\beta_{0}+\sum_{j=1}^{M}\beta_{j}E_{j}(l)+\varepsilon , & j=1\cdots M \end{array}$$

Therefore, an imputed complete dataset ${\hat{G}}_{1}$ is generated, which will be used by KNN or LLS to estimate ${\hat{G}}_{2}(l,i)$, where the Euclidean distance *d*_*m *_between the target gene **g**_*i *_and the neighbor genes **g**_*m *_(*m *= 1 ⋯ *R*) are computed and *k *nearest genes are selected for imputation. Therefore in this case, all the genes including those initially have missing values will be used to compute the distance *d *'*s*, which contributes a lot to the selection of the neighbor genes. The missing value estimated by KNN is [16]

(12) $${\hat{G}}_{2}(l,i)=\sum_{m=1}^{k}g_{m}(l)/d_{m}/\sum_{m=1}^{k}1/d_{m}$$

and the LLS estimation ${\hat{G}}_{2}(l,i)$ is [20]

(13) $${\hat{G}}_{2}(l,i)=b^{T}{\left(N^{T}\right)}^{\text{†}}W_{l}$$

where **N **is the matrix formulation of neighbor genes deleting the *l-*th component, **b **is a *k*-dimensional vector consisting of the *l*-th component of neighbor genes, **w**_*l *_is non-missing entries of **g**_*i*_.

Finally, the missing value $\hat{G}(l,i)$ was filled using,

(14) $$\hat{G}(l,i)=\lambda {\hat{G}}_{1}(l,i)+(1-\lambda ){\hat{G}}_{2}(l,i)$$

where the weight parameter *λ *is a positive value between 0 and 1. The weight *λ *= 0 means the pattern expressions associated with acetylation patterns contributes to the final imputation by providing an initial imputed complete dataset to the conventional LLS or KNN. The weight *λ *= 1 means the missing values were imputed totally by using the pattern expressions associated with acetylation patterns. The value of *λ *was determined by a training procedure (See Results).

In addition, an iterative procedure was integrated into HAI method to increase the imputation accuracy. The iterative imputation algorithm was originally suggested by Rich Caruana and executed by the following two steps [54]. On the first step, missing values are estimated from observed values. On the second step, the accuracy of fill-in values will be reused to improve the imputation through recursive process. This iterative procedure was executed repeatedly until the differences between newly updated values and previous values converge. Since in most of our experimental simulations, nearly all the imputed values were converged within less than 3 iterations, we did 3 iterations for the accuracy comparison. However, the empirical convergence of the algorithm remains to be theoretical formulated.

## Authors' contributions

QX designed the study, implemented the algorithms, carried out the experiments, analyzed the results and drafted the manuscript. XD participated in the design of the study, analysis of the results and drafting of the manuscript. YD participated in the implement of some algorithms. CH read the manuscript and gave suggestions. JW participated in the gene clustering. JF and ZD participated in the analysis and discussion. All authors read and approved the final manuscript.

## Supplementary Material

Additional file 1 **Selection of neighbourhood size *k *for KNN, GOKNN and knnHAI**. The neighbourhood size *k *of KNN, GOKNN and knnHAI was determined by selecting *k *value at which KNN obtained the smallest NRMSE. The horizontal axis is the varying range of *k *from 5 to 40. The vertical axis is NRMSE of 50 independent and random test runs. We observed that 10 neighbours were enough for nearly all of the datasets at different percentages, thus the value *k *= 10 was used in each test run. Click here for file

Additional file 2 **Selection of neighbourhood size *k *for LLS, GOLLS and llsHAI**. The neighbourhood size *k *of LLS, GOLLS and llsHAI was determined by select *k *value at which LLS obtained the smallest NRMSE. The horizontal axis is the varying range of *k *from 60 to 200. The vertical axis is NRMSE of 50 independent and random test runs. We observed that 150 neighbours were enough for nearly all of the datasets at different percentages, thus the value *k *= 150 was used in each test run. Click here for file

Additional file 3 **Selection of parameter *λ *for knnHAI**. The parameter *λ *of knnHAI was determined by select *λ *value with which knnHAI obtained the smallest NRMSE. The horizontal axis is the varying range of *λ *from 0 to 1. The vertical axis is NRMSE of 50 independent and random test runs. We observed that the optimal *λ *value greatly depends on the dataset under investigation. Generally the optimal *λ *values are much larger in datasets of Sp.elutriation, Sp.alpha and Calcineurin. The optimal values of *λ *are very similar in non-time series datasets of Gasch and Histone. While for Diauxic, the optimal *λ *is very small, which suggests the number of conditions has a marked influence [13]. Therefore, we select different *λ *for each dataset in each test run of knnHAI. Click here for file

Additional file 4 **Selection of parameter *λ *for llsHAI**. The parameter *λ *of llsHAI was determined by select *λ *value with which llsHAI obtained the smallest NRMSE. The legends are the same as Additional file 3. We observed that the optimal *λ *value also greatly depends on the dataset under investigation. It seems that the optimal *λ *values for datasets of Sp.elutriation, Sp.alpha, Diauxic and Calcineurin are very small. While the optimal values of *λ *in non-time series datasets of Gasch and Histone are much larger. Therefore, we select different *λ *for each dataset in each test run of llsHAI. Click here for file

Additional file 5 **Influence of the missing value models**. Comparisons of NRMSE performances for KNN, LLS, GOKNN, GOLLS, knnHAI, and llsHAI in six datasets. The parameters *λ *are optimized by using the random model, while the burst models are used in the testing phase. The legends are the same as Figure 1. There are no significant differences between the performance of HAIimpute methods in this case with those in Figure 2. This suggests that the HAIimpute method is robust even if we do not know the underlying missing value models. Click here for file

Additional file 6 **Influence of used histone acetylation patterns**. The two panels on the left are the comparisons of the NRMSE performances for KNN, LLS and knnHAI, llsHAI using ORF acetylation patterns. The horizontal axis is the varying quantity of used ORF acetylation patterns from 0 to 68. The two panels on the right are the comparisons of the NRMSE performances for KNN, LLS and knnHAI, llsHAI using IGR acetylation patterns. The horizontal axis is the varying quantity of used IGR acetylation patterns from 0 to 53. The vertical axis is NRMSE of 50 independent and random test runs for each method. The missing value percentages of 10%, 15% and 20% are used in the test runs. The dataset of SP.elu was used here. Click here for file

Additional file 7 **NRMSE performance with respect to Iterative process**. Comparisons of the NRMSE performances for llsHAI and knnHAI with different number of iterations. The horizontal axis is the varying number of iterations from 0 to 4. The vertical axis is NRMSE of 50 independent and random test runs for each method. The datasets of SP.alpha and Calcineurin were used here. Click here for file
