# Supplementary figures and images for: Missing value imputation for microarray gene expression data using histone acetylation information
Source: BMC Bioinformatics. 2008 May 29;9:252. doi: 10.1186/1471-2105-9-252 (PMC2432074; doi:10.1186/1471-2105-9-252)

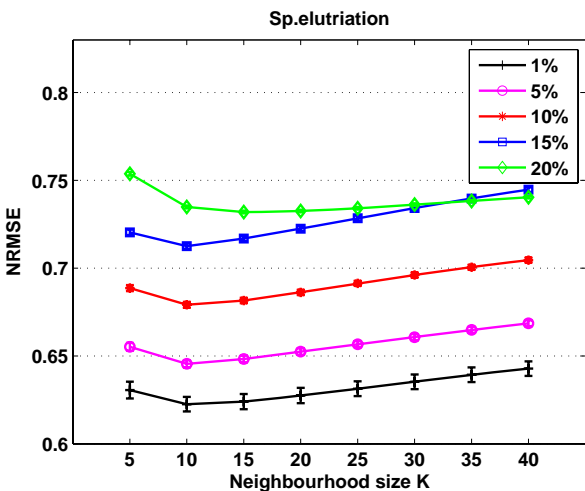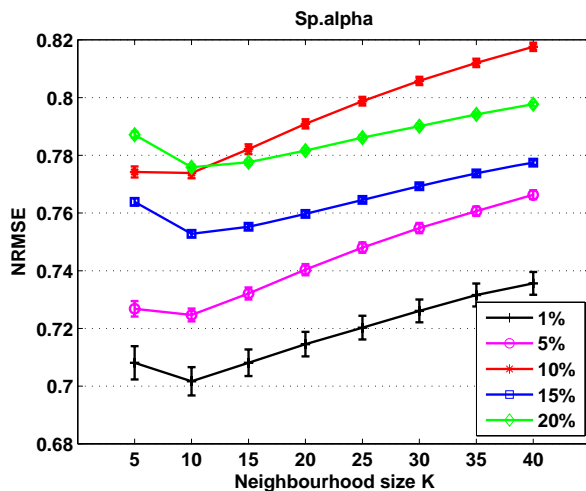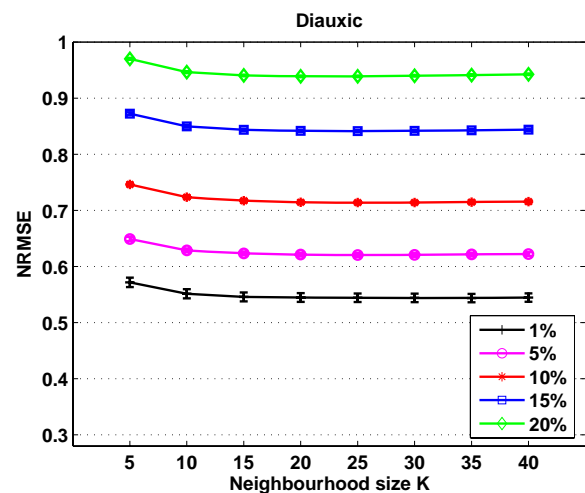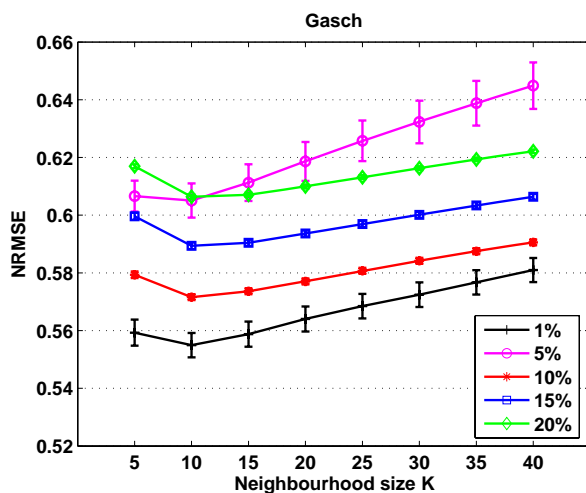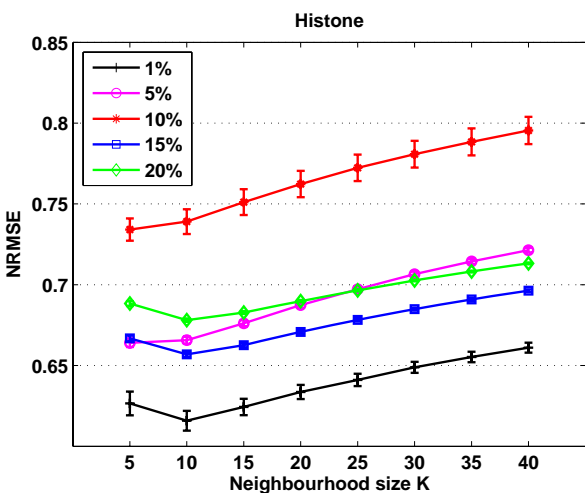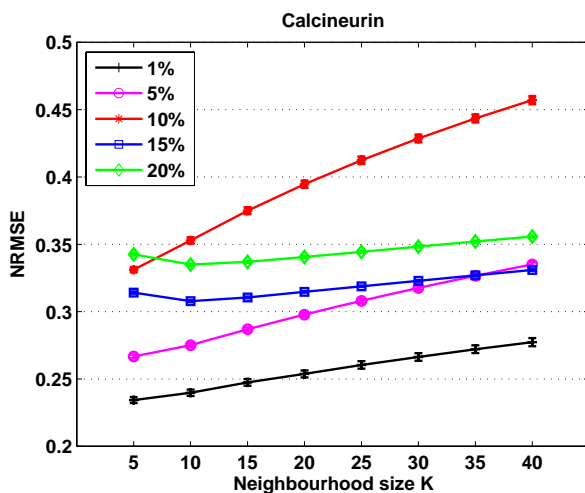

Supplement: Additional file 1 — Selection of neighbourhood size k for KNN, GOKNN and knnHAI. The neighbourhood size k of KNN, GOKNN and knnHAI was determined by selecting k value at which KNN obtained the smallest NRMSE. The horizontal axis is the varying range of k from 5 to 40. The vertical axis is NRMSE of 50 independent and random test runs. We observed that 10 neighbours were enough for nearly all of the datasets at different percentages, thus the value k = 10 was used in each test run. [file 1471-2105-9-252-S1.pdf]

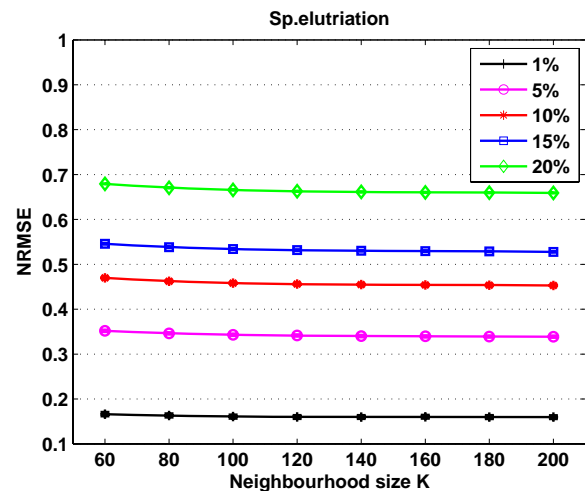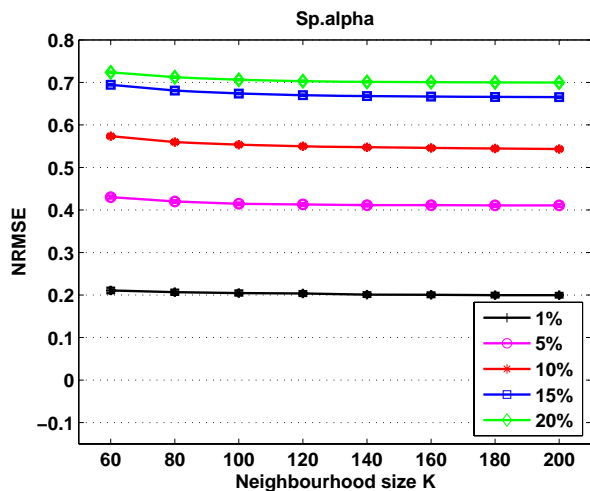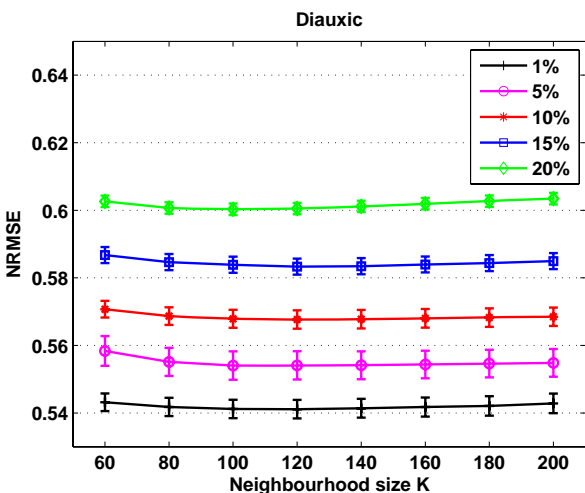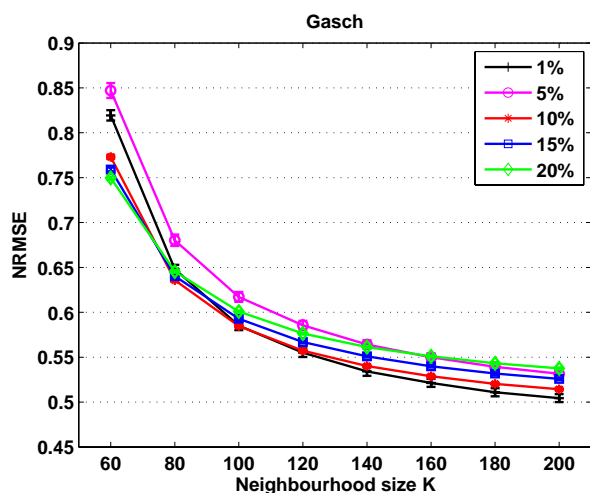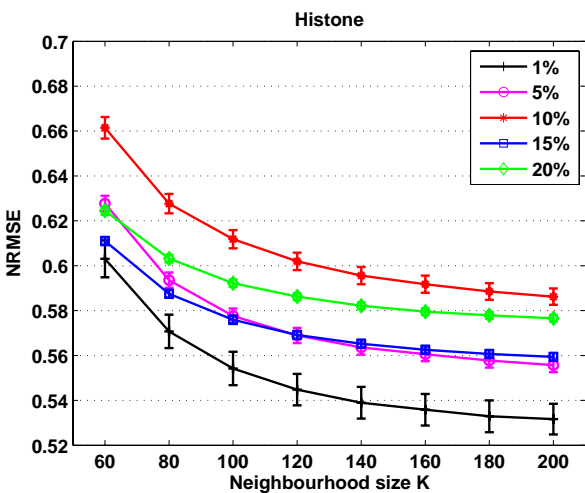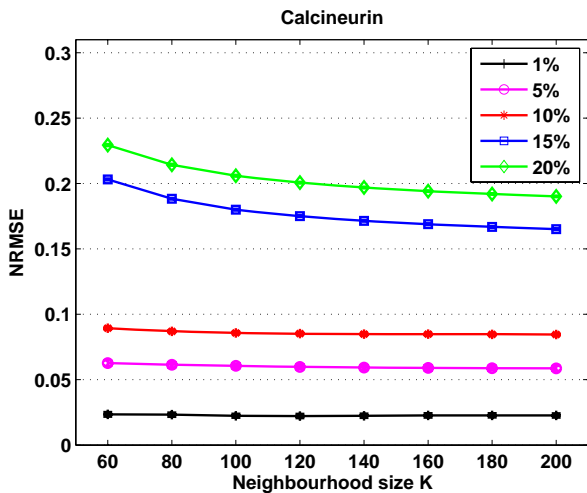

Supplement: Additional file 2 — Selection of neighbourhood size k for LLS, GOLLS and llsHAI. The neighbourhood size k of LLS, GOLLS and llsHAI was determined by select k value at which LLS obtained the smallest NRMSE. The horizontal axis is the varying range of k from 60 to 200. The vertical axis is NRMSE of 50 independent and random test runs. We observed that 150 neighbours were enough for nearly all of the datasets at different percentages, thus the value k = 150 was used in each test run. [file 1471-2105-9-252-S2.pdf]

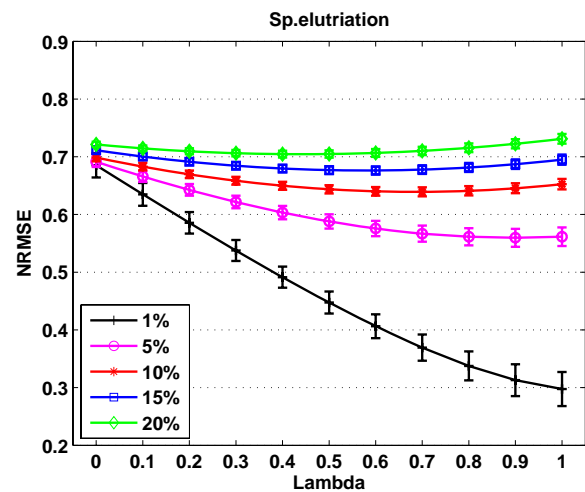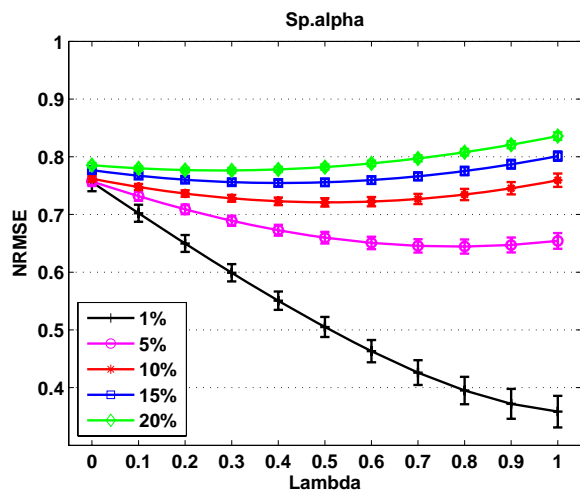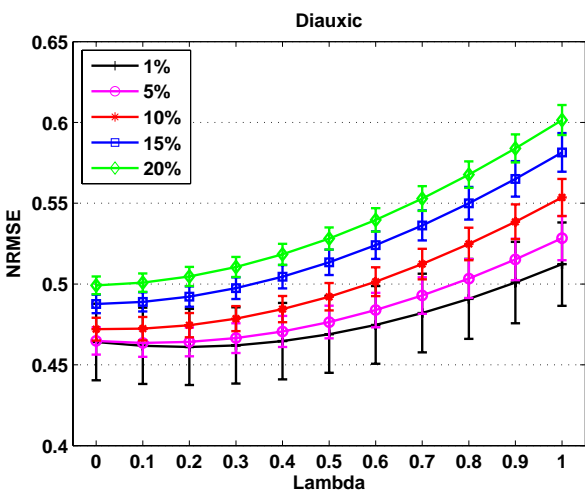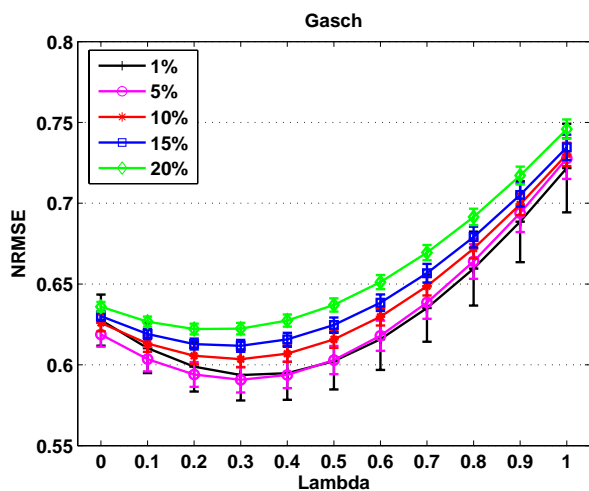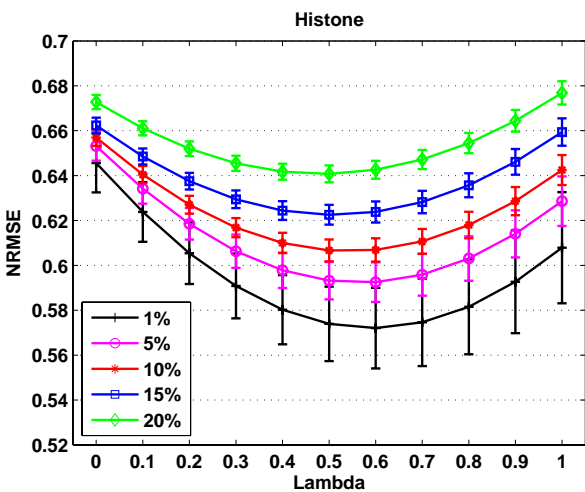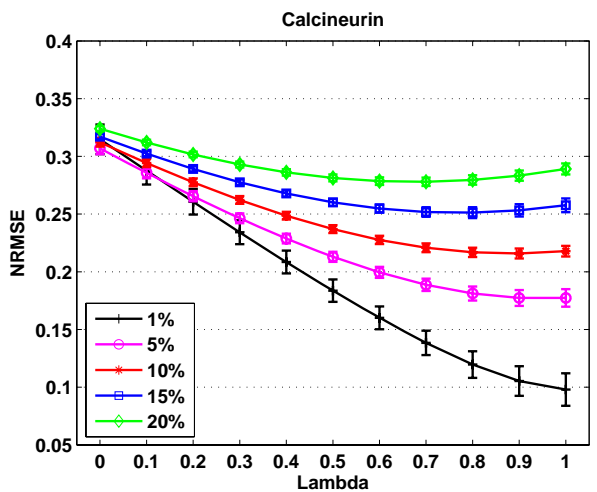

Supplement: Additional file 3 — Selection of parameter λ for knnHAI. The parameter λ of knnHAI was determined by select λ value with which knnHAI obtained the smallest NRMSE. The horizontal axis is the varying range of λ from 0 to 1. The vertical axis is NRMSE of 50 independent and random test runs. We observed that the optimal λ value greatly depends on the dataset under investigation. Generally the optimal λ values are much larger in datasets of Sp.elutriation, Sp.alpha and Calcineurin. The optimal values of λ are very similar in non-time series datasets of Gasch and Histone. While for Diauxic, the optimal λ is very small, which suggests the number of conditions has a marked influence [13]. Therefore, we select different λ for each dataset in each test run of knnHAI. [file 1471-2105-9-252-S3.pdf]

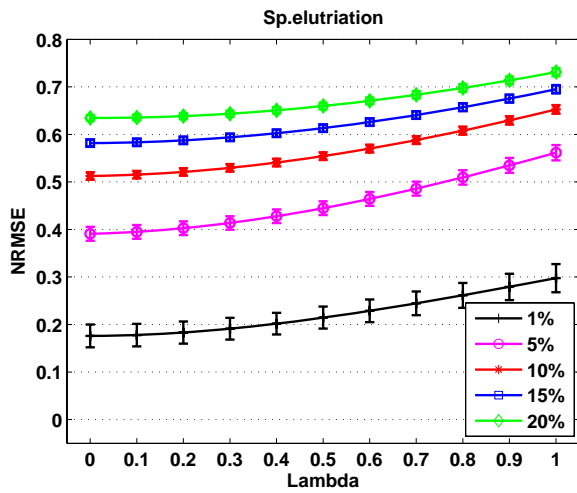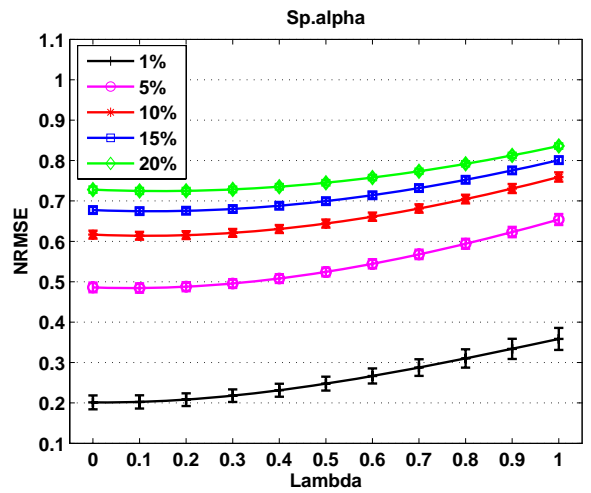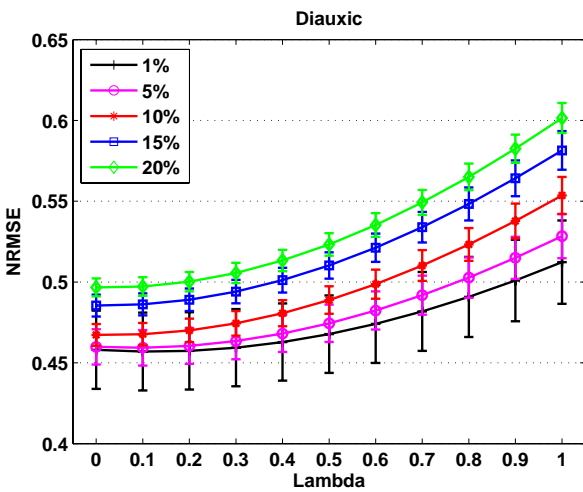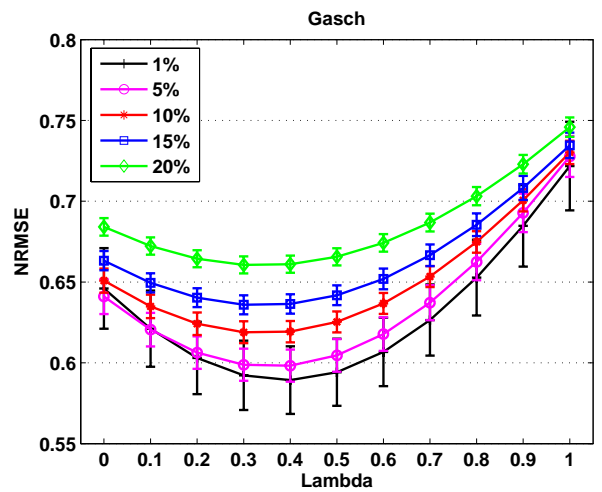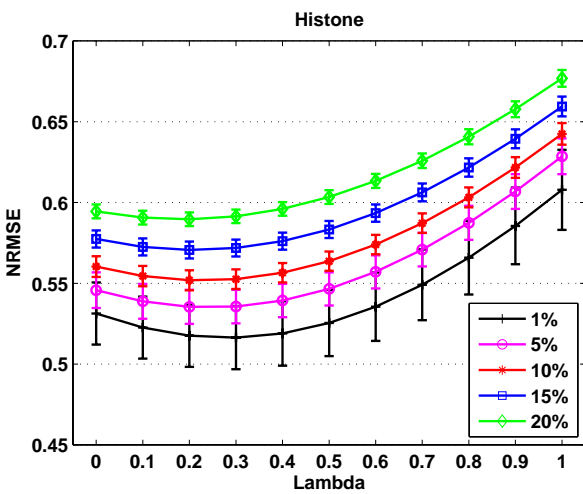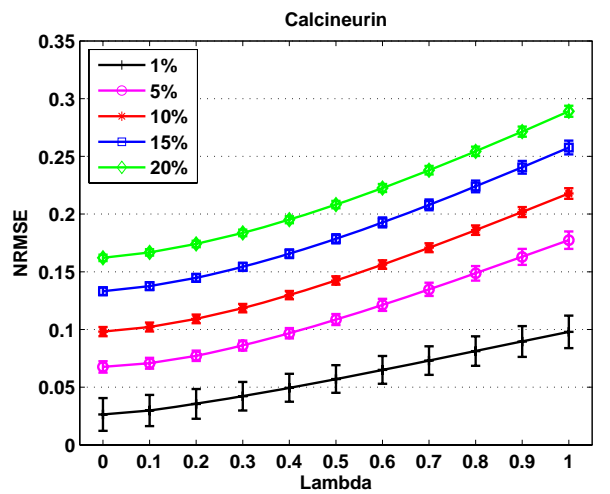

Supplement: Additional file 4 — Selection of parameter λ for llsHAI. The parameter λ of llsHAI was determined by select λ value with which llsHAI obtained the smallest NRMSE. The legends are the same as Additional file 3. We observed that the optimal λ value also greatly depends on the dataset under investigation. It seems that the optimal λ values for datasets of Sp.elutriation, Sp.alpha, Diauxic and Calcineurin are very small. While the optimal values of λ in non-time series datasets of Gasch and Histone are much larger. Therefore, we select different λ for each dataset in each test run of llsHAI. [file 1471-2105-9-252-S4.pdf]

Sp.elutriation

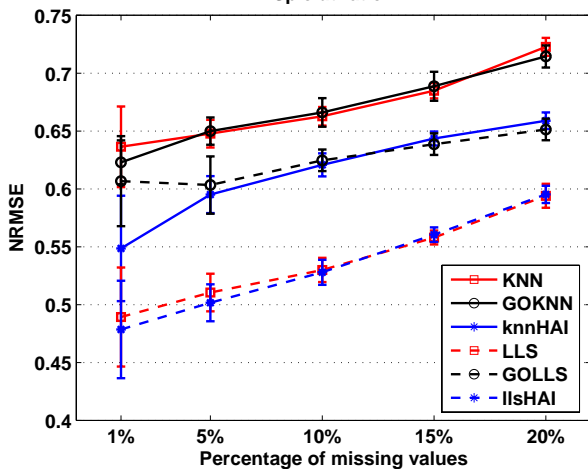

Sp.alpha

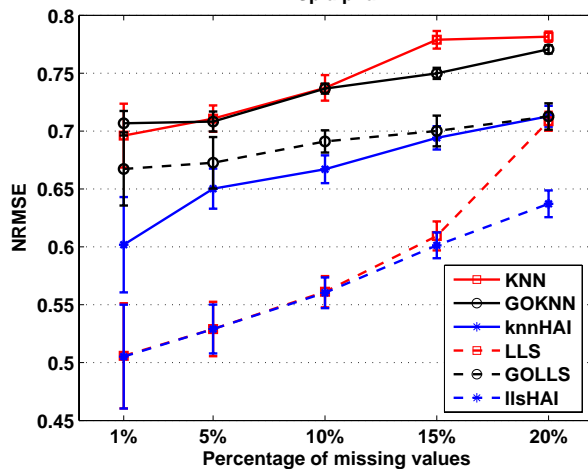

Diauxic

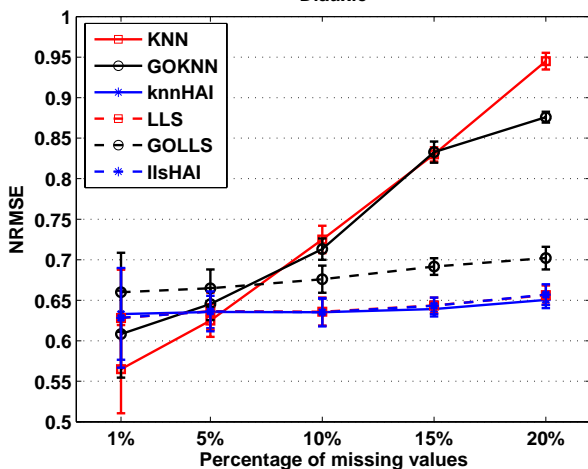

Gasch

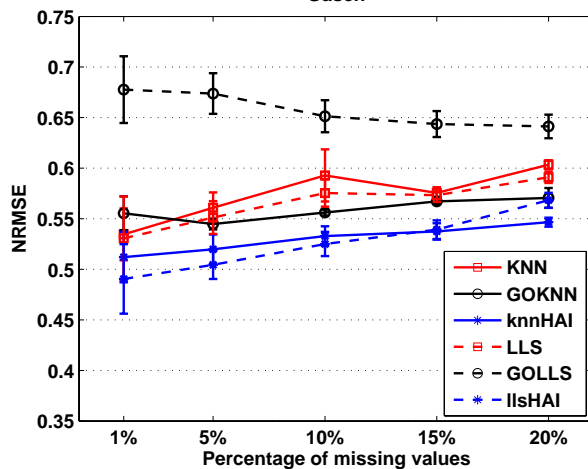

Histone

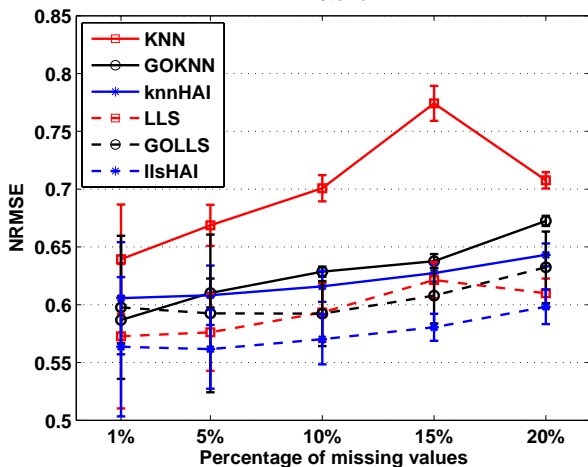

Calcineurin

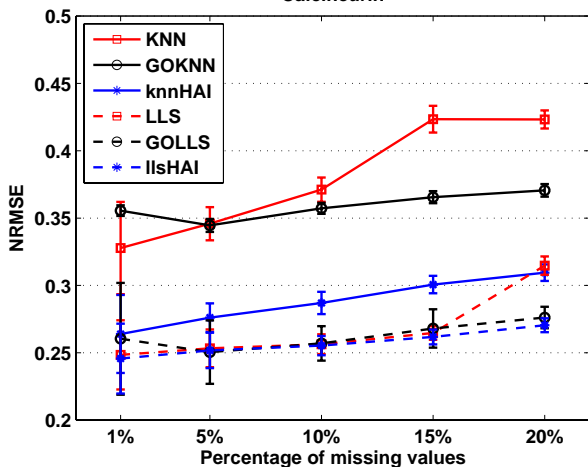

Supplement: Additional file 5 — Influence of the missing value models. Comparisons of NRMSE performances for KNN, LLS, GOKNN, GOLLS, knnHAI, and llsHAI in six datasets. The parameters λ are optimized by using the random model, while the burst models are used in the testing phase. The legends are the same as Figure 1. There are no significant differences between the performance of HAIimpute methods in this case with those in Figure 2. This suggests that the HAIimpute method is robust even if we do not know the underlying missing value models. [file 1471-2105-9-252-S5.pdf]

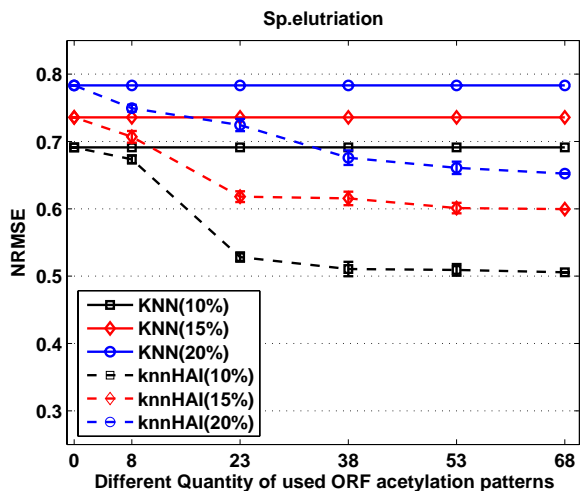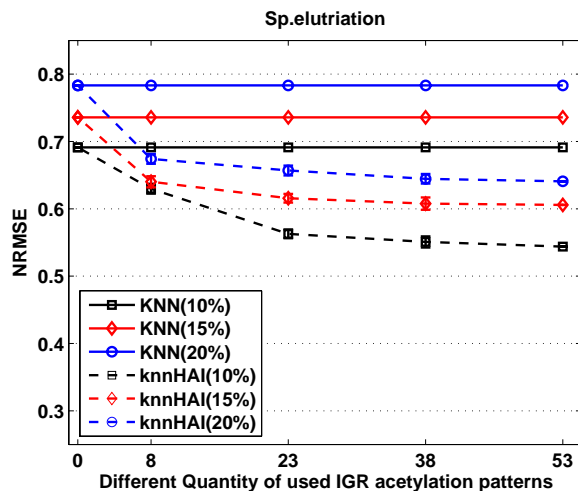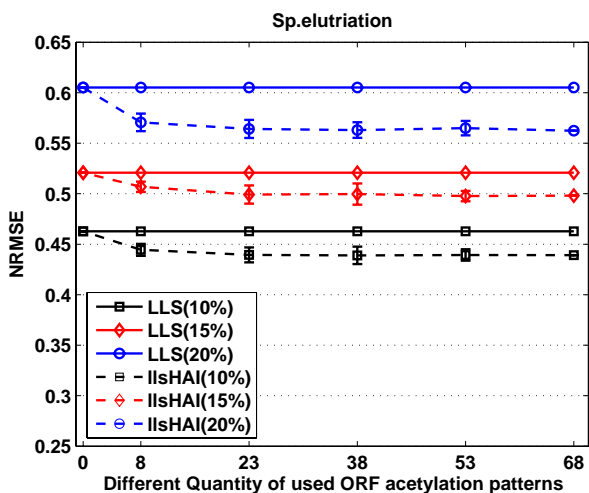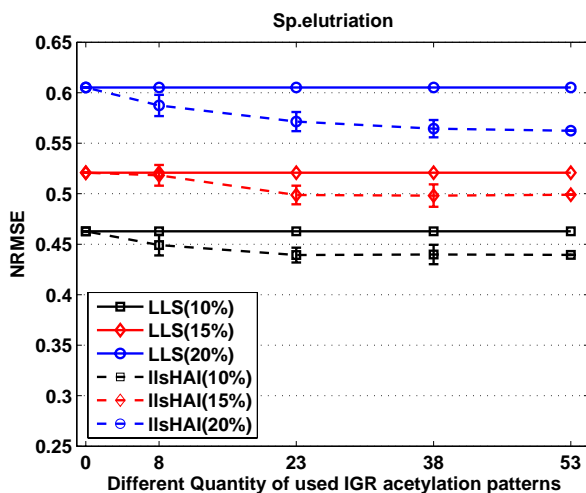

Supplement: Additional file 6 — Influence of used histone acetylation patterns. The two panels on the left are the comparisons of the NRMSE performances for KNN, LLS and knnHAI, llsHAI using ORF acetylation patterns. The horizontal axis is the varying quantity of used ORF acetylation patterns from 0 to 68. The two panels on the right are the comparisons of the NRMSE performances for KNN, LLS and knnHAI, llsHAI using IGR acetylation patterns. The horizontal axis is the varying quantity of used IGR acetylation patterns from 0 to 53. The vertical axis is NRMSE of 50 independent and random test runs for each method. The missing value percentages of 10%, 15% and 20% are used in the test runs. The dataset of SP.elu was used here. [file 1471-2105-9-252-S6.pdf]

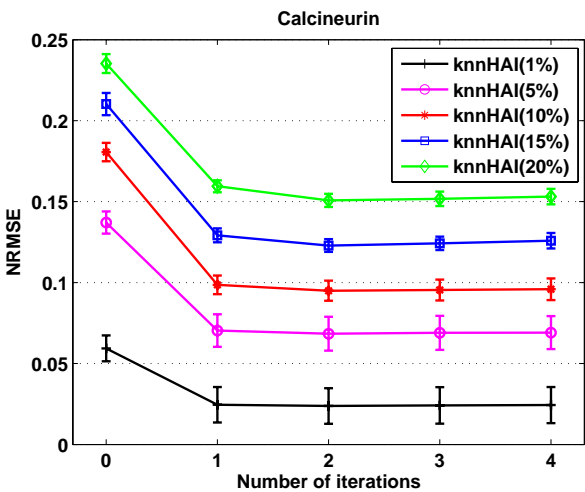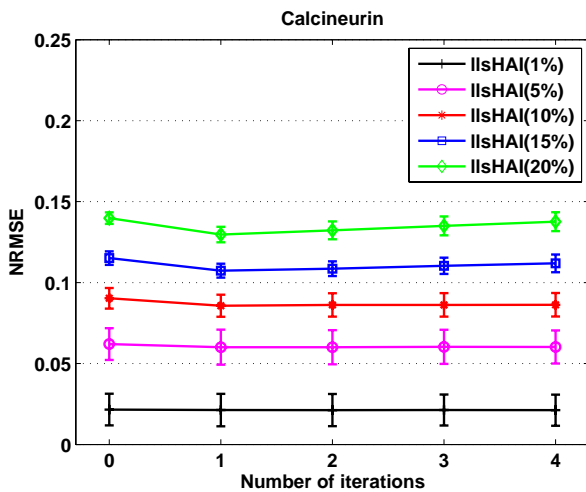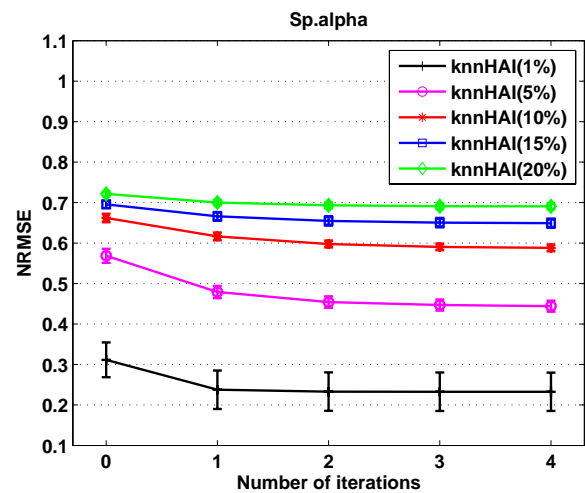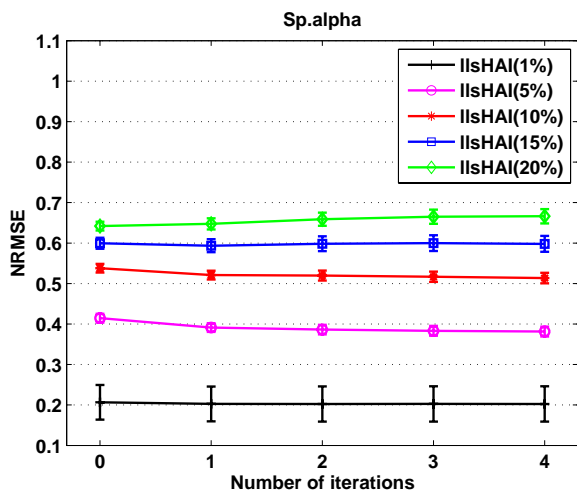

Supplement: Additional file 7 — NRMSE performance with respect to Iterative process. Comparisons of the NRMSE performances for llsHAI and knnHAI with different number of iterations. The horizontal axis is the varying number of iterations from 0 to 4. The vertical axis is NRMSE of 50 independent and random test runs for each method. The datasets of SP.alpha and Calcineurin were used here. [file 1471-2105-9-252-S7.pdf]
